# MULTIPLE ENRICHMENT TOOLS POINT TO A CENTRAL ROLE OF CARDIAC MUSCLE IN EXTREME LONGEVITY

**DOI:** 10.1093/geroni/igad104.2244

**Published:** 2023-12-21

**Authors:** Svetlana Ukraintseva, Deqing Wu, Hongzhe Duan, Konstantin Arbeev, Anatoliy Yashin

**Affiliations:** Duke University, Durham, North Carolina, United States; Social Science Research Institute, Duke University, Durham, North Carolina, United States; Social Science Research Institute, Duke University, Durham, North Carolina, United States; Social Science Research Institute, Duke University, Durham, North Carolina, United States; Social Science Research Institute, Duke University, Durham, North Carolina, United States

## Abstract

Few genes showed consistent positive associations with extreme longevity in humans. We conducted genome-wide association study of various parental lifespans, LS (85+, 90+, 95+, 100+ vs. 75-), using UK Biobank data. At ages 100+, the negative effect of APOE4 on LS diminished, while SNPs with positive associations with LS emerged among the top results, including rare variants. One of the top SNPs (rs756319938) was in CDKN1A (P21) gene involved in cell senescence, and its rare variant (protein truncating) was significantly (P-value< 10-7) overrepresented in extreme longevity cases. Such nonsense mutations generally result in non-functional protein. In case of P21, this might contribute to longevity via better regenerative response. The latter was previously demonstrated in mice that lacked the p21 expression. For SNPs that were associated with LS>=100 with P-value < 10-4, we identified ~400 corresponding genes and run multiple pathway/process enrichment analyses using different tools utilizing various resources (GO, KEGG, OMIM, MSigDB, and others), to identify pathways/processes that show up across most analyses. We found that cardiac muscle function was among the top pathways/processes yielded by majority of the enrichment tools. In conclusion, we found new rare and common SNPs that were positively associated with extreme longevity of mothers and fathers of UKB participants, and investigated relevant biological pathways. Results of multiple enrichment tools converged on a central role of cardiac muscle function in extreme longevity, while separate enrichment tools did not allow to see this picture. Our results imply that cardiac regeneration is a plausible target for interventions into longevity limits.

